# Synthesis of metal iodates from an energetic salt†

**DOI:** 10.1039/d0ra02250k

**Published:** 2020-04-07

**Authors:** I. Shancita, Kelsea K. Miller, Preston D. Silverstein, Joseph Kalman, Michelle L. Pantoya

**Affiliations:** Department of Mechanical Engineering, Texas Tech University Lubbock TX 79409 USA michelle.pantoya@ttu.edu; Mechanical and Aerospace Engineering Department, California State University Long Beach CA 90840 USA

## Abstract

Iodine containing oxidizers are especially effective for neutralizing spore forming bacteria by generating iodine gas as a long-lived bactericide. Metal iodates have been shown to be strong oxidizers when combined with aluminum fuel particles for energy generating applications. One method to produce metal iodates *in situ* is by using metal oxides and an energetic salt: aluminum iodate hexahydrate (Al(H_2_O)_6_(IO_3_)_3_(HIO_3_)_2_), which is called AIH. In this study, the thermal stability and reactivity of AIH with metal oxides commonly used in energetic formulations was investigated. Three metal oxides: bismuth(iii) oxide (Bi_2_O_3_), copper(ii) oxide (CuO), and iron(iii) oxide (Fe_2_O_3_) were investigated because of their different oxygen release properties. Each metal oxide powder was combined with AIH powder. Thermal stability and reactivity were characterized by differential scanning calorimetry (DSC) and thermogravimetric analysis (TG) and reactive properties calculated to supplement experimental observations. Powder X-ray diffraction (XRD) was also used to identify the product species at various stages of heating corresponding to exothermic activity. Results show that AIH decomposition is entirely endothermic but, with the addition of metal oxide powder to AIH, exothermic reactions transform metal oxides into more stable metal iodates. This analysis provides an understanding of the compatibility of AIH with metal oxides and contributes to the development of novel energetic composites that have the advantages of both thermal and biocidal mechanisms for spore neutralization.

## Abstract 2

Iodine containing oxidizers are especially effective for neutralizing spore forming bacteria by generating iodine gas as a long-lived bactericide.

## Graphical abstract

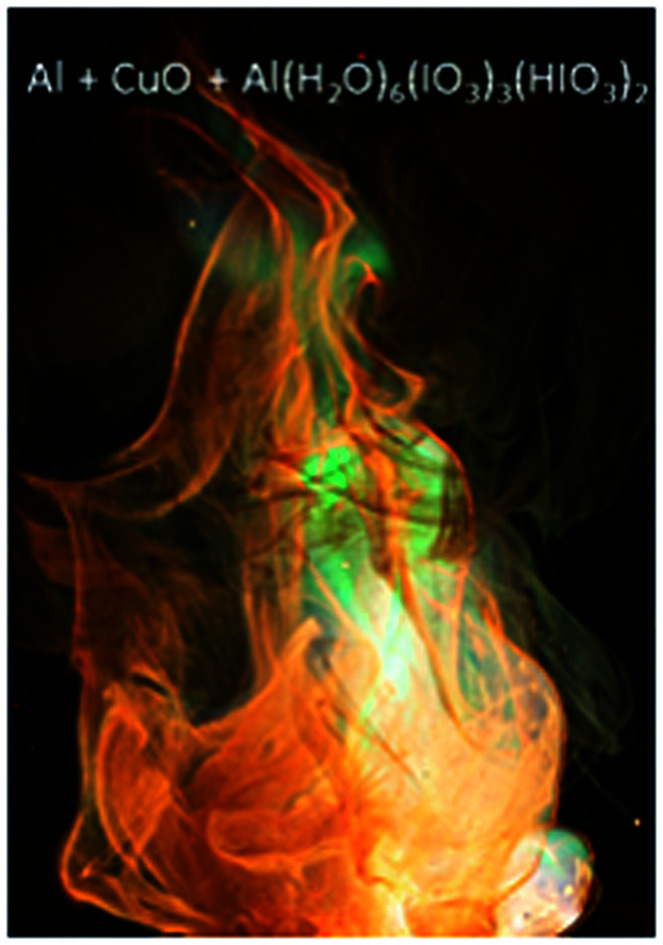

## Introduction

Aluminium nanoparticles (nAl) are passivated by a thin amorphous aluminium oxide (Al_2_O_3_) shell 4–5 nm thick. Smith *et al.*^1^ recently transformed the Al_2_O_3_ shell on nAl particles into (Al(H_2_O)_6_(IO_3_)_3_(HIO_3_)_2_), which is called aluminium iodate hexahydrate (AIH) using a wet chemistry approach. Gottfried *et al.*^2^ showed that AIH coated nAl particles have the potential to react at time scales relevant to a detonation event. The potential for AIH coated Al particles to produce fast energy release rates has motivated further investigation of this over-oxidized energetic salt: AIH.

A wet chemistry approach was used to synthesize AIH on the surface of nAl particles by immersing nAl particles in an iodic acid (HIO_3_) solution. The ensuing reaction between the Al_2_O_3_ shell and HIO_3_ solution formed AIH through a proposed polarization reaction mechanism.^3^ The AIH coated nAl particles are stable under standard atmospheric conditions and can be combined with other reactants with the potential of producing high energy release rates during Al oxidation owing to the low oxygen release temperature of AIH (*i.e.*, ∼150 °C (ref. 2)) that more readily exposes the Al core for oxidation compared to an Al_2_O_3_ passivation.

Pure AIH powder was also synthesized from aluminium hydroxide (Al(OH)_3_) immersed in an iodic acid (HIO_3_) solution and the wet chemistry synthesis is described in detail in Kalman *et al.*^4^ The crystal structure for AIH includes Al surrounded by a six-member hydration ring, [Al(H_2_O)_6_]^3+^. The IO_3_^−^ in the solution bonds with outer hydration ring, [Al(H_2_O)_6_]^3+^ to balance the charge and form Al(H_2_O)_6_(IO_3_)_3_(HIO_3_)_2_. Gottfried *et al.*^2^ showed the molecular structure has an oxygen balance of at least 17.7%.^2^ The AIH powder was used by Kalman *et al.*^4^ as an oxidizer when combined with nAl powder. Even as a discretely separate oxidizer (as opposed to a passivation shell), AIH exhibited exceptionally fast energy propagation rates (*i.e.*, >1000 m s^−1^) when combined with nAl.^4^ The over oxidized nature of AIH coupled with low oxygen release temperature and high gas generation properties (*i.e.*, products include water vapour, oxygen, and iodine gas) all contribute towards its favourable energetic performance.

Not only does AIH enhance the energy release rate of metal fuel particles, but AIH also contains a high concentration of iodine which is an important biocidal agent for neutralizing spore forming bacteria such as bacillus anthracis.^5^ In fact, three metal iodates: Fe(IO_3_)_3_, Cu(IO_3_)_2_, and Bi(IO_3_)_3_ were combined with Al fuel particles and all three formulations exhibited orders of magnitude higher energy release behaviour compared to their metal oxide counterpart.^6^ These three metal iodates were shown to be strong oxidizers with aluminium (Al) particles and react to yield very exothermic and violent reactions.^7^ Therefore, forming metal iodates *in situ* reaction with AIH would also provide multiple strong oxidizers in the reaction zone. Creating *in situ* metal iodates during reaction using AIH and metal oxides could be an effective approach to designing reactant formulations that decompose to allow multiple pathways for oxygen to react with the fuel and release molecular iodine.

The objective of this study was to use AIH to synthesize metal iodates that are strong oxidizers for energetic material applications but also release iodine gas upon reaction thereby enabling technologies that have biocidal applications. This thermal and chemical analysis was a fundamental first step toward understanding the reaction dynamics associated with AIH relative to energetic formulations. The objective was accomplished through differential scanning calorimetry, thermogravimetric analysis, and powder X-ray diffraction studies that were supplemented with analytical calculations that provided insight to the synthetic pathways observed experimentally.

## Experimental

### Synthesis of AIH

**Fig. 1:**
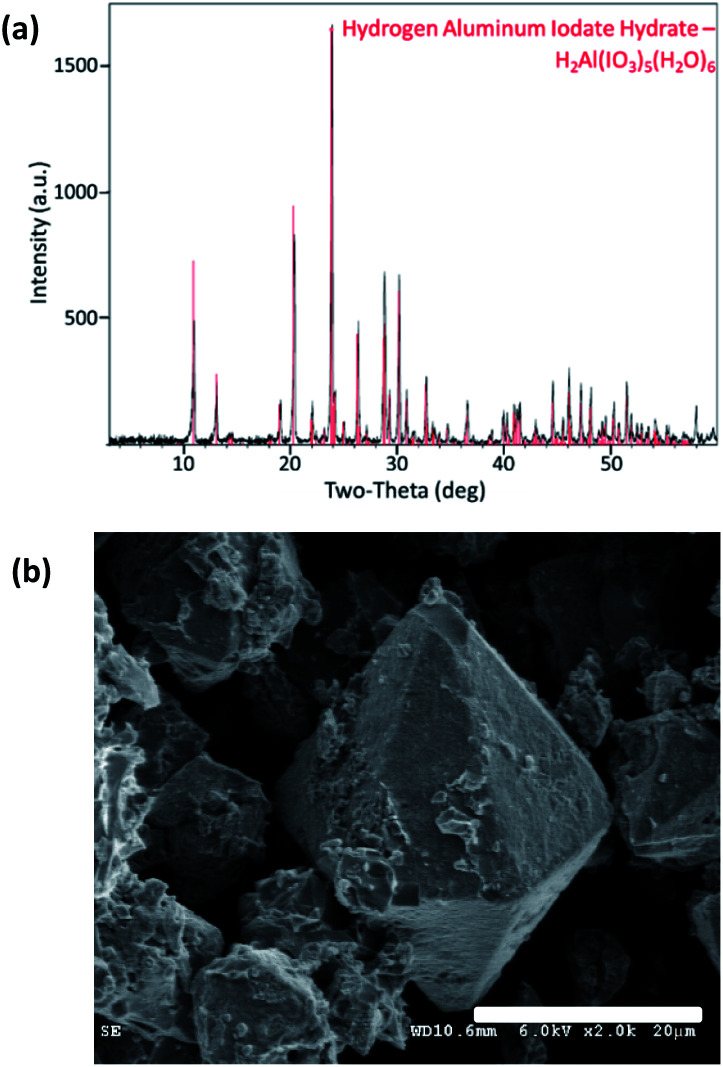
(a) Diffraction pattern from PXRD measurement indicating pure AIH powder (the red lines indicate the AIH reference overlaps the measured peaks). Data was collected from 0–90° 2*θ* with parallel beam geometry in continuous *θ*–2*θ* mode with a collection time of 2° min^−1^ and a step size of 0.02°. (b) SEM image depicting hexagonal pyramidal geometries of pure AIH crystals. Note 20 μm scale bar for reference. Image was taken with a Hitachi S-4300 high resolution field emission SEM at an accelerating voltage of 6 kV.

Pure Al(H_2_O)_6_(IO_3_)_3_(HIO_3_)_2_ powder was synthesized *via* wet chemistry using bayerite, α-Al(OH)_3,_ as the precursor and is described by Kalman *et al.*^4^ but summarized here. Commercial iodine pentoxide (I_2_O_5_) powder (Sigma Aldrich, Saint Louis, MO) was dissolved in water using a magnetic stirrer to form an iodic acid (HIO_3_) solution. The α-Al(OH)_3_, supplied by Sigma Aldrich, was added to the HIO_3_ solution and mixed at an elevated temperature until the solution was clear. Acetonitrile (ACN) was then added when α-Al(OH)_3_ appeared to have completely dissolved and the solution was cooled to room temperature. The ACN facilitates Al(H_2_O)_6_(IO_3_)_3_(HIO_3_)_2_ crystal growth large enough for filtration from the solution. The rinsed and collected crystals were confirmed 100% Al(H_2_O)_6_(IO_3_)_3_(HIO_3_)_2_ purity by PXRD analysis shown in Fig. 1a. Particle size was analysed using an APE Aerosizer particle size analyser (TSI Incorporated), and was measured to have a specific surface area of 0.14 m^2^ g^−1^. The surface morphology of AIH powder (Al(H_2_O)_6_(IO_3_)_3_(HIO_3_)_2_) is seen *via* scanning electron microscopy (SEM) images shown in Fig. 1b, and crystals have hexagonal pyramidal geometries.

The metal oxide powders have spherical morphology with average particle diameters of 90–210 nm for Bi_2_O_3_, <50 nm for CuO, and <50 nm for Fe_2_O_3_ and all powders were provided by Sigma Aldrich (St. Louis, MO). These precursor metal oxides were analysed with PXRD (described below) to show that CuO is 100% pure, whereas Fe_2_O_3_ and Bi_2_O_3_ include other phases: Fe_2_O_3_ is 73.9 wt% Fe_2_O_3_, 14.1 wt% Fe_3_O_4_, and 12 wt% amorphous material; and, Bi_2_O_3_ is 94.7 wt% α-Bi_2_O_3_ (monoclinic) and 5.3 wt% Bi_2_(CO_3_)O_2_.

All powders were prepared in batches of 50 mg, with 25 mg of AIH and 25 mg of metal oxide powder. The mass percentage of AIH and metal oxide was the controlled variable and remained constant, and the molar percentages varied according to Table 1. The powders were mixed using isopropanol as the process control agent (PCA). The slurry was mixed in a 100 ml beaker with a magnetic stirrer for 5 min at 400 rpm, then poured into a Pyrex™ plate and placed in a fume hood to evaporate the PCA so that the solid powder mixture could be reclaimed.

**Table 1:** Molar percentages of the mixtures

Mixtures	Mole percentage (%)	
AIH + CuO	AIH	CuO
	7.3	92.7
AIH + Fe_2_O_3_	AIH	Fe_2_O_3_
	13.63	86.37
AIH + Bi_2_O_3_	AIH	Bi_2_O_3_
	31.54	68.46

### Material characterization

Differential scanning calorimetry (DSC) and thermogravimetric analysis (TGA) were performed using a NETZSCH STA 449 F3 Jupiter simultaneous thermal analyser (STA). In each experiment, approximately 5–7 mg of mixture was loaded in an alumina crucible and heated from 30 to 800 °C at a heating rate of 10°C min^−1^ in a nitrogen environment.

Powder XRD was performed by a Rigaku MiniFlex II powder diffractometer to measure the product species from the DSC and TGA experiments as well as the pure reactant materials using Bragg–Brentano geometry. The X-ray diffraction patterns were obtained by scanning a 2*θ* range of 3–70° in continuous mode with a step size of 0.02° and a scan time of 1.0 min per degree, unless otherwise indicated as in Fig. 1a. The X-ray source was Cu Kα radiation (*λ* = 1.5418 Å) with an anode voltage of 30 kV and a current of 15 mA. The diffraction intensities were recorded with a D/teX Ultra position sensitive detector. The samples were prepared as a powder mount on a zero-background holder and the diffraction patterns were processed with the software JADE v9.1. The amorphous content was determined by including an amorphous hump (pseudo-Voigt) into the Rietveld calculation.

## Results

**Fig. 2:**
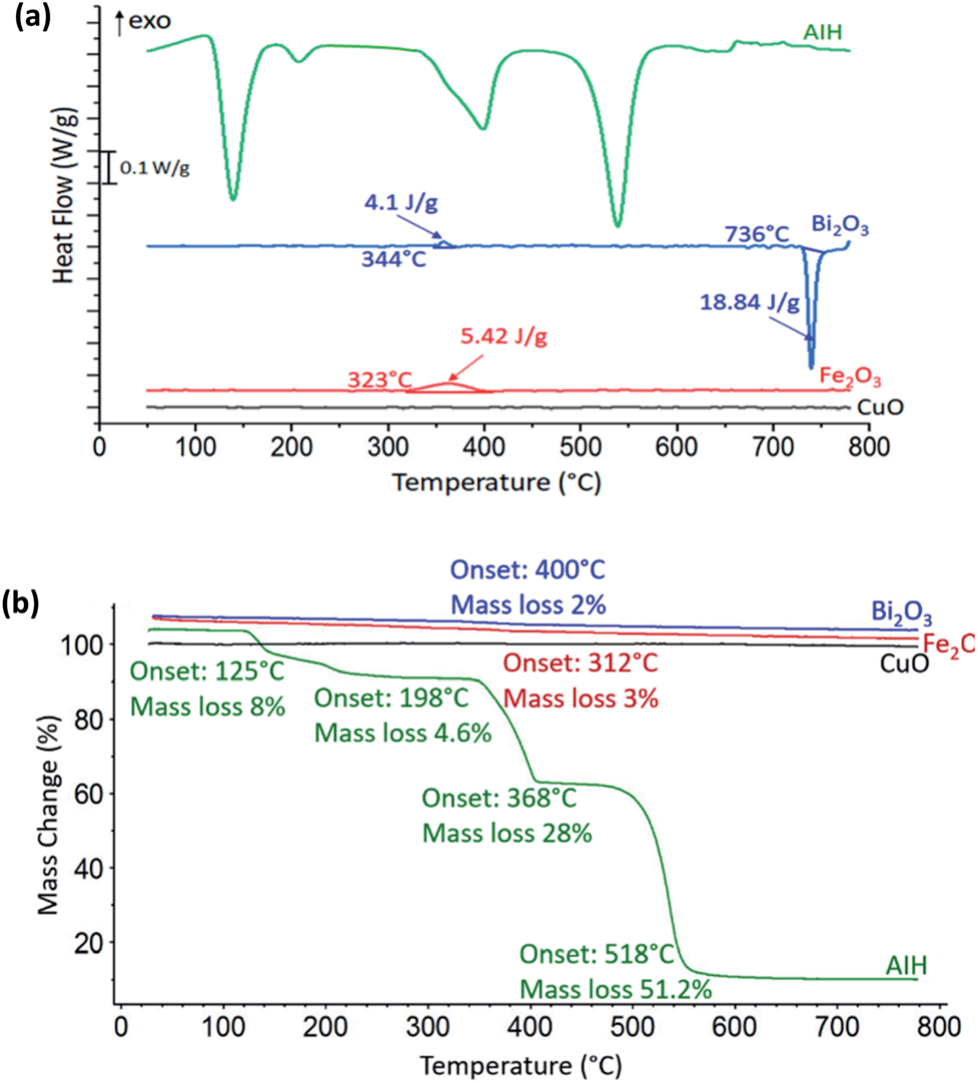
(a) Heat flow as a function of temperature for CuO (black), Fe_2_O_3_ (red), Bi_2_O_3_ (blue), and AIH (green) at 10°C min^−1^ heating rate in nitrogen environment. Note the scaling for heat flow is indicated on the graphic and onset temperature and enthalpy are also indicated. (b) Corresponding mass loss percentage curves with onset temperature and mass loss percentages indicated.

In Fig. 2a heat flow curves are shown as a function of temperature for the individual materials with the corresponding mass loss curves shown in Fig. 2b. For CuO, there is no observable exothermic or endothermic reaction, whereas there is one exothermic reaction for Fe_2_O_3_ that corresponds to a decomposition process that releases oxygen from Fe_2_O_3_ to Fe_3_O_4_ (ref. 8) with onset temperature at 323 °C in Fig. 2a and we observed a slight gradual mass loss of 3 wt% with onset temperature at 312 °C in Fig. 2b. For Bi_2_O_3_ in Fig. 2a, the endotherm with onset temperature at 736 °C is rapid phase transition from α-Bi_2_O_3_ to δ-Bi_2_O_3_ which is a metastable phase of Bi_2_O_3_.^9^ There is one small exothermic reaction with onset temperature at 344 °C which corresponds to the presence of both mixed α-and β-phase of Bi_2_O_3_,^9^ which is confirmed by the PXRD results of 96 wt% of α-Bi_2_O_3_ and 4 wt% of β-Bi_2_O_3_. There is a corresponding mass loss of 2 wt% with onset temperature at 400 °C from Fig. 2b.

Reaction 1 (R1) shows condensed phase decomposition stages for AIH and provided so that the mass loss trend in Fig. 2b can be analysed further.R1
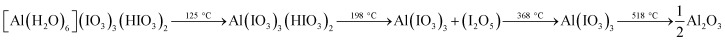


Fig. 2b shows 92% mass loss from AIH decomposition overall which corresponds well with the predicted mass loss from (R1) of 95%. The good correlation suggests that the mechanism described by (R1) is likely the condensed phase decomposition stages observed experimentally. The deviations from (R1) may be caused by ancillary reactions that are an artefact of hydration, gas generation, and mass loss including iodine and oxygen gas.

Fig. 2b shows AIH begins to decompose by dehydration of hydroxyls surrounding Al, and transition of HIO_3_ to HI_3_O_8_ both with onset temperature at 125 °C as indicated by the first endotherm and described in (R1). The second endotherm with onset temperature at 198 °C is dehydration of HI_3_O_8_ to I_2_O_5_. Then, I_2_O_5_ starts to decompose at 368 °C and AIH becomes pure anhydrous aluminium iodate (Al(IO_3_)_3_) (see (R1)). The final endotherm indicates decomposition of Al(IO_3_)_3_ to Al_2_O_3_ that started at 518 °C.^4^ It is noted that a similar decomposition mechanism for AIH is described by Kalman *et al.*^4^

**Fig. 3:**
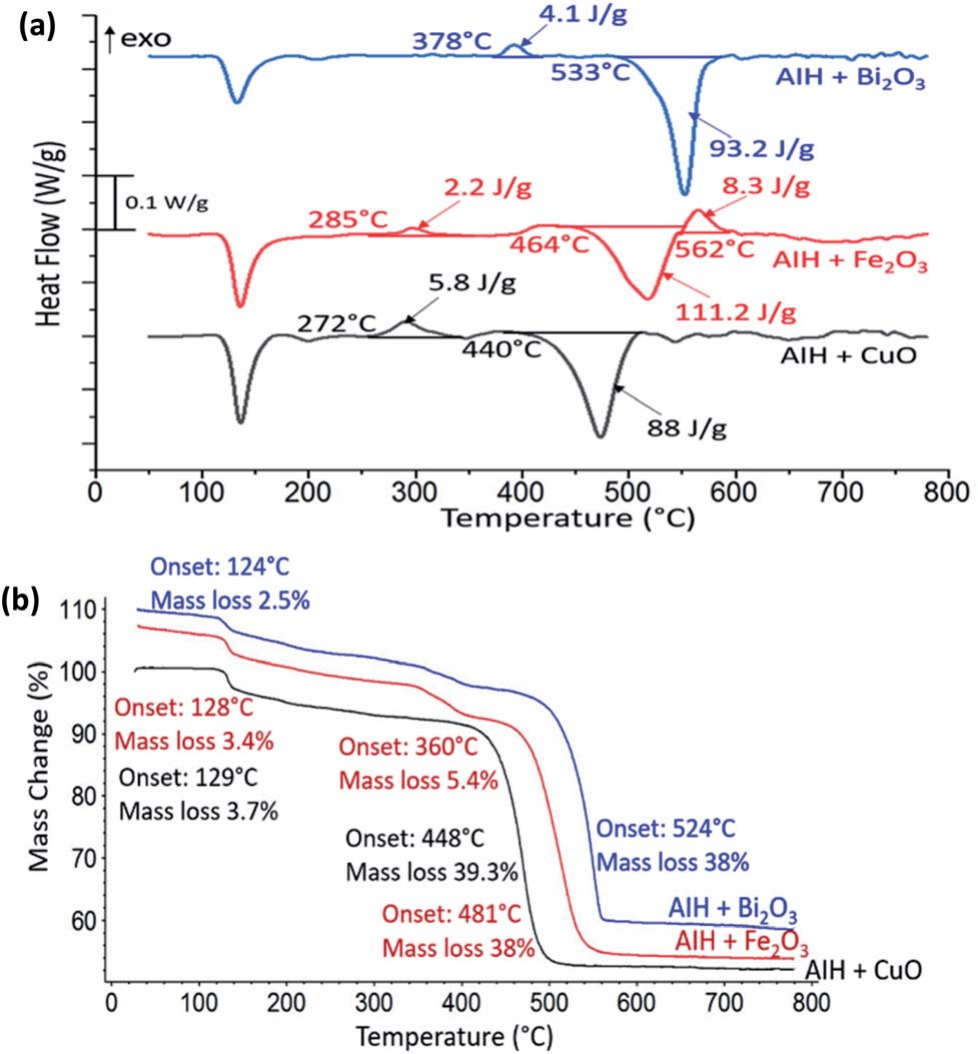
(a) Heat flow as a function of temperature for AIH + CuO (black), AIH + Fe_2_O_3_ (red) and AIH + Bi_2_O_3_ (blue) in a nitrogen environment. Onset temperatures and enthalpies are indicated. (b) Corresponding mass loss percentage curves with onset temperatures and mass loss percentages indicated.

In Fig. 3a the heat flow curves for AIH + CuO, AIH + Fe_2_O_3_ and AIH + Bi_2_O_3_ as a function of temperature are shown. For all mixtures, endothermic phase change and exothermic reactions are observed. The corresponding mass loss curves are shown in Fig. 3b. The first mass loss for all mixtures corresponds to AIH dehydration and transformation of HIO_3_ to HI_3_O_8_ similar to Fig. 2b. Total mass loss corresponds to 43 wt%, 47 wt% and 41 wt% for AIH + CuO, AIH + Fe_2_O_3_ and AIH + Bi_2_O_3_, respectively.

In Fig. 3a for AIH + CuO, one exothermic reaction is observed with onset temperature at 272 °C and specific enthalpy of 5.8 J g^−1^ and is complete by 360 °C. The onset temperature for this exothermic reaction is much lower than the oxygen release temperature of CuO at 527 °C according to one study^10^ and between 700–900 °C in another^11^ suggesting condensed phase CuO participates in the exothermic reaction. Table 2 shows the PXRD analysis that identifies the wt% of product species from annealed reactants at 360 °C, *i.e.*, at the completion of the exothermic peak shown in Fig. 3a and at a higher temperature (800 °C). Table 2 indicates the exothermic reaction may be formation of copper iodate (Cu(IO_3_)_2_). The PXRD patterns of the product species for AIH + CuO annealed at 360 °C and 800 °C are shown in ESI Fig. S1.†

**Table 2:** Product species from AIH + CuO annealed at indicated temperatures

Phase ID	Materials (wt%)	
	AIH + CuO	
	Annealed 360 °C	Annealed 800 °C
CuO	46.4	24.3
Cu(IO_3_)_2_	18.5	—
Al(IO_3_)_3_	29.7	—
Amorphous	5.4	75.7

For AIH + Fe_2_O_3_, the exothermic reaction in Fig. 3a has an onset temperature of 285 °C, specific enthalpy of 2.2 J g^−1^, and is complete at 360 °C. The onset temperature is lower than the oxygen release temperature of Fe_2_O_3_ at 827 °C according to one study,^10^ but there is a possibility of decomposition of Fe_2_O_3_ to Fe_3_O_4_ due to oxygen release when heated between 128–481 °C,^8^ which indicates that the exothermic reaction could occur at the lower oxygen release temperature for Fe_2_O_3_ conversion to Fe_3_O_4_. Also, Fe_2_O_3_ + AIH shows additionally 5.4% mass loss at 360 °C shown in Fig. 3b which could coincide with oxygen loss from the reaction. The second exothermic reaction with onset temperature at 562 °C of specific enthalpy of 8.3 J g^−1^ may correspond to further conversion of Fe_2_O_3_ to amorphous Fe_3_O_4_ that has been reported to occur exothermically at elevated temperatures.^12^Table 3 establishes the presence of Fe_3_O_4_ in AIH + Fe_2_O_3_ that was heated to 360 °C and formation of iron iodate (Fe(IO_3_)_3_) as well as Al(IO_3_)_3_. Fig. S2 in ESI† shows the PXRD patterns of the product species for AIH + Fe_2_O_3_ annealed at 360 °C and 800 °C.

**Table 3:** Product species from AIH + Fe_2_O_3_ annealed at indicated temperatures

Phase ID	Materials (wt%)	
	AIH + Fe_2_O_3_	
	Annealed 360 °C	Annealed 800 °C
Fe_2_O_3_	—	15.7
Fe_3_O_4_	38.1	—
Fe(IO_3_)_3_	27.9	—
Al(IO_3_)_3_	15.6	—
Amorphous	18.4	84.3

For AIH + Bi_2_O_3_, the exothermic reaction is observed at a higher onset temperature than the other reactions (at 378 °C), with a specific enthalpy of 4.1 J g^−1^ and is completed by 420 °C. The onset of reaction is at much lower temperatures (*i.e.*, 378 °C) than its oxygen release temperature (*i.e.*, 1347 °C) and in the condensed phase like the Bi_2_O_3_ reaction with Al.^9,13^Table 4 shows the product species after the first exothermic reaction includes bismuth oxide iodate (BiO(IO_3_)) and Al(IO_3_)_3_. Fig. S3 in ESI† shows the PXRD patterns of the product species for AIH + Bi_2_O_3_ annealed at 420 °C and 800 °C.

**Table 4:** Product species from AIH + Bi_2_O_3_ annealed at indicated temperatures

Phase ID	Materials (wt%)	
	AIH + Bi_2_O_3_	
	Annealed 420 °C	Annealed 800 °C
β-Bi_2_O_3_	19.4	—
BiO(IO_3_)	70.4	—
Al(IO_3_)_3_	10.2	—
Al_2_O_3_		18.1
Others		81.9

**Fig. 4:**
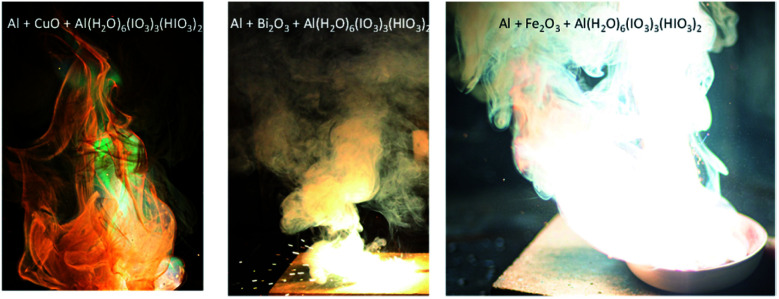
Photographs of self-sustained reactions of aluminium fuel powder combined with the binary oxidizer combinations indicated. Note the copper oxide mixture produces green colour flames, bismuth oxide mixture shows particles ejected from the flame, and the iron oxide mixture produces bright white flames. Photographs were taken from a cell-phone camera.

While the binary combinations of AIH and metal oxides are too fuel lean to ignite and react, adding aluminium (Al) fuel powder to each of these binary oxidizer mixtures created self-sustained reactions. Fig. 4 shows photographs of mixtures of Al, AIH, metal-oxide (*e.g.*, CuO, Bi_2_O_3_, or Fe_2_O_3_), combined with a silicone-based binder. Mixture were prepared to demonstrate the potential for the binary oxidizers to produce exothermic and self-sustained reactions. The images are qualitative representations of reactivity.

## Discussion

Fig. 2 and 3 show decomposition of AIH begins at 119 °C due to dehydration of AIH and decomposition of HIO_3_ to HI_3_O_8_. Fig. 3a shows exothermic reactions occur for all mixtures and start between 272–378 °C, after the initial stages of AIH decomposition. Tables 2–4 show that at 360 °C, Cu(IO_3_)_2_, Fe(IO_3_)_3_,and Al(IO_3_)_3_ are the main products for respective reactions and at the higher temperature of 420 °C, BiO(IO_3_) forms as well as Al(IO_3_)_3_. From molar ratios in Table 1 and the results in Tables 2–4 neglecting amorphous content, global reactions for the first annealing temperature can be described in (R2)–(R4).R2
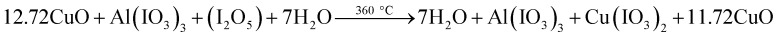
R3
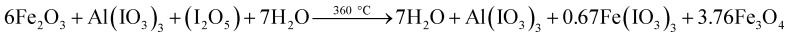
R4
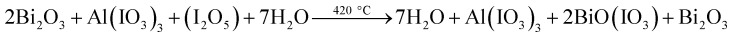


The weight percent of product species predicted from (R2)–(R4) are compared to the actual weight percent of product species measured from Tables 2–4 and a summary of the results are shown in Table 5.

**Table 5:** Predicted and measured wt% of product species for AIH + metal oxides annealed at 360 or 420 °C as indicated in (R2)–(R4). Amorphous is Am

Mix	Predicted wt% of product species from (R2)–(R4)			Measured wt% of product species from powder XRD analysis			
AIH + CuO	CuO	Cu(IO_3_)_2_	Al(IO_3_)_3_	CuO	Cu(IO_3_)_2_	Al(IO_3_)_3_	Am
	49	22	29	46	19	30	5
AIH + Fe_2_O_3_	Fe_3_O_4_	Fe(IO_3_)_3_	Al(IO_3_)_3_	Fe_3_O_4_	Fe(IO_3_)_3_	Al(IO_3_)_3_	Am
	48	22	30	38	28	16	18
AIH + Bi_2_O_3_	Bi_2_O_3_	BiO(IO_3_)	Al(IO_3_)_3_	Bi_2_O_3_		BiO(IO_3_)	Al(IO_3_) _3_
	26	44	30	19		70	10

The predicted and measured product concentrations are similar for CuO + AIH but there is greater disparity in the comparison for Fe_2_O_3_ + AIH and Bi_2_O_3_ + AIH reactions. For Fe_2_O_3_ + AIH, less Fe_3_O_4_ and Al(IO_3_)_3_ is formed than predicted but Fe_3_O_4_ has been reported in an amorphous phase^14^ and may account for the amorphous concentration indicated in Table 5. The deviation between predicted and measured oxygen concentrations may be an artefact of the difficulties in tracing the abundant oxygen reactions that are a part of every molecule involved in the reaction and most of the possible ancillary reactions also involve a transfer of oxygen. Also, the higher temperature of the Bi_2_O_3_ + AIH exothermic reaction may facilitate dissociation reactions that include iodine and oxygen gas generation that may escape the reaction zone and reduce the Al(IO_3_)_3_ concentration thereby increasing the BiO(IO_3_) concentration.

Table 6 shows the predicted (*i.e.*, from (R2)–(R4)) and measured concentrations (*i.e.*, from Tables 2–4) of metal oxides that reacted with the iodate species from AIH. The comparison reveals that the measured concentration of metal oxides that reacted is much higher for each reaction than the predicted concentration. The greater consumption of metal oxides especially seen for the Fe_2_O_3_ + AIH and Bi_2_O_3_ + AIH reactions may contribute to the correspondingly higher production of metal iodate species measured in Table 5 compared with predicted concentrations, indicating that the kinetics forming metal iodates are more favoured than (R3) and (R4) predict.

**Table 6:** Predicted and measured weight percentage of metal oxide reacted in each reaction

Mixtures	Predicted wt% of metal oxide reacted	Actual wt% of metal oxide reacted
AIH + CuO	CuO	CuO
	2	7.2
AIH + Fe_2_O_3_	Fe_2_O_3_	Fe_2_O_3_
	4	23.8
AIH + Bi_2_O_3_	Bi_2_O_3_	Bi_2_O_3_
	48	61.2

According to (R2)–(R4), the most likely contribution to exothermic energy comes from the following reactions shown in (R5)–(R7) that form metal iodates by consuming iodine pentoxide.

R5 12.72CuO + I_2_O_5_ → Cu(IO_3_)_2_ + 11.72CuO

R6 6Fe_2_O_3_ + I_2_O_5_ → 0.67Fe(IO_3_)_3_ + 3.76Fe_3_O_4_

R7 2Bi_2_O_3_ + I_2_O_5_ → 2BIO(IO_3_) + Bi_2_O_3_

R8 2Cu(IO_3_)_2_ → 2CuO + 2I_2_ + 2O_2_

R9 4Fe(IO_3_)_3_ → 2Fe_2_O_3_ + 6I_2_ + 15O_2_

R10 4BiO(IO_3_) → 2Bi_2_O_3_ + 2I_2_ + 5O_2_

R11 4Al(IO_3_)_3_ → 2Al_2_O_3_ + 6I_2_ + 15O_2_

The following decomposition of formed metal iodates is highly endothermic and occurs at higher temperatures (*i.e.*, 400–600 °C) as shown in Fig. 3a and can be described by reaction (R8)–(R10) with the decomposition of Al(IO_3_)_3_ shown in (R11).

Heats of formation for the metal iodates are not experimentally reported, thus theoretical estimates were adopted.^15^ The data used for the heat of reaction and decomposition calculations are summarized in Table 7.

**Table 7:** Enthalpy of formation, reaction, and decomposition of metal oxides and iodates. Reference for each value is noted as a superscript

Species	Enthalpy of formation (kJ mol^−1^)	Enthalpy of reaction (J g^−1^)	Enthalpy of decomposition (J g^−1^)
CuO	−183.7 ^15^	(R5): −14.2	—
Fe_2_O_3_	−866.45 ^15^	(R6): 237.1	—
Fe_2_O_3_	−810.5 ^15^	(R6): −22.7	
Fe_3_O_4_	−1242.71 ^15^		
Bi_2_O_3_	−786.85 ^15^	(R7): −109.85	—
Al_2_O_3_	−1660.5 ^15^		
I_2_O_5_	−372.45 ^15^		
Cu(IO_3_)_2_	−724.23 ^15^	—	(R8): 1458.64
Fe(IO_3_)_3_	−1106.3 ^15^	—	(R9): 1368.8
BiO(IO_3_)	−844.27 ^15^	—	(R10): 903.86
Al(IO_3_)_3_	−1651.91 ^15^	—	(R11): 1659.01
I_2_	62.4 ^16^	—	—
O_2_	0 ^16^	—	—

The enthalpy of reaction calculation for (R5) is −14.2 J g^−1^ (Table 7) and the best agreement to the experimentally measured value of −5.8 J g^−1^. The enthalpy of reaction for (R7) is more exothermic than the experimental data. However, for reaction (R6), by considering different crystallographic space groups for formation enthalpy, there are both endothermic and exothermic reaction enthalpies ranging from −22.7 to 237.1 J g^−1^ (Table 7) which are not consistent with our experimental data. The discrepancies between calculated and measured values may be attributed to multiple reasons. For example, the amorphous content is not accounted for in the calculations which was 5 and 18%, by weight for the CuO and Fe_2_O_3_ cases, respectively and iron oxides have been observed to be amorphous at similar conditions.^14^ Also, the data from ref. 15 are estimates and reported values for the enthalpy of formation for I_2_O_5_ vary by orders of magnitude in the literature.^17^ These differences and variations in formation enthalpy based upon different crystallographic space groups will influence the overall reaction enthalpy. For example, using iron oxide *P*4_1_2_1_2 (tetragonal) and *Cmcm* (orthorhombic) results in reaction enthalpy ranging from 237.1 J g^−1^ to −22.4 J g^−1^ for (R6). Similar differences may be present for CuO. Many metal iodate enthalpies of formation have not been measured or reported in the literature. In summary, all three reactions (R5)–(R7) can produce exothermic behaviour that agree with the experimental data (Fig. 3a) and are attributed to metal iodate formation.

Following the exothermic reactions in Fig. 3a, endotherms are observed at higher temperatures (*i.e.*, >400 °C) that correspond with the decomposition of metal iodates formed in (R5)–(R7)^7^ as well as Al(IO_3_)_3_. There is crystal phase transition for Bi(IO_3_)_3_ between 290–375 °C which could account for the BiO(IO_3_) measured here and the decomposition of BiO(IO_3_) begins at 400 °C,^6,7^ and is comparable to the measured temperature from Fig. 3a at 533 °C. The measured endotherms compare well with the calculated enthalpies from the decomposition reactions (Table 7) and are consistent with other^7^ reporting of metal iodate decomposition behaviour, confirming the formation and subsequent decomposition of the metal iodates. Similar calculations as described for the exothermic reactions were conducted for these endothermic reactions. The enthalpies of reaction calculated for (R8)–(R11) are shown in Table 7, and the calculated results are much more endothermic than the experimental values. Tables 2–4 show that the 800 °C annealed concentrations (*i.e.* after the endothermic reactions) are primarily amorphous which limits the comparison.

All decomposition stages of AIH are endothermic (see Fig. 2a). Overall, the presence of metal oxides affects the decomposition of AIH by exothermically reacting with iodine oxide species formed upon AIH decomposition at temperatures greater than 250 °C (see (R5)–(R7)). In another study by Kalman *et al.*,^4^ Al particles were added to AIH particles and two exothermic reactions were observed: the first was 100.7 J g^−1^ with an onset of 274 °C while the second was 893.8 J g^−1^ with an onset of 549 °C. Kalman *et al.*^4^ proposed the first exothermic reaction was attributed to reactions between Al_2_O_3_ and iodine oxide species resulting from AIH decomposition. The Al_2_O_3_ present in their mixture was an inherent part of the Al particles, encapsulating the nano-scale Al particles and passivating the pyrophoric Al core. The first exothermic reaction was called a pre-ignition reaction (PIR) and described as surface reactions between Al_2_O_3_ passivating Al particles with iodine oxide species resulting from AIH decomposition at temperatures > 250 °C. Therefore, Al_2_O_3_ may produce a similar exothermic reaction with iodine oxide species as described in (R5)–(R7) because a similar exothermic reaction was observed by Kalman *et al.*^4^

This analysis shows that AIH can be combined with metal oxides to synthesize *in situ* reaction metal iodates. The formation of metal iodates at these relatively low temperatures (<450 °C) would create multiple reaction pathways for oxidation reactions with metal fuel particles like Al, as demonstrated in Wang *et al.*^7^ Because metal iodates form at temperatures lower than the melting and ignition temperature of Al particles (*i.e.*, 660 °C), multiple metal iodates would provide immediate and direct interaction with Al particles to enable several kinetic routes for fuel particle oxidation. Future work will explore the potential of using metal oxides in mixtures of AIH and fuel particles.

## Conclusions

Aluminium iodate hexahydrate Al(H_2_O)_6_(IO_3_)_3_(HIO_3_)_2_, named AIH, is entirely endothermic upon decomposition in DSC analysis. However, when AIH is combined with a metal oxide, AIH decomposition species are reactive with metal oxides. This study examined AIH combined with copper oxide (CuO), iron oxide (Fe_2_O_3_) and bismuth trioxide (Bi_2_O_3_) and their exothermic reaction with iodine oxide species decomposed from AIH produced the corresponding metal iodate. Experimental results confirm the formation of Cu(IO_3_)_2_, Fe(IO_3_)_3_, BiO(IO_3_), and Al(IO_3_)_3_ at temperatures in the range of 360 °C to 420 °C and show that the metal iodate is more stable than the metal oxide. The exothermic activity is at lower temperatures than the metal oxide oxygen release temperatures suggesting multiphase reactions between gaseous decomposition species from AIH and condensed phase metal oxides. Thermochemical calculations confirm the exothermic formation of Cu(IO_3_)_2_, Fe(IO_3_)_3_, and BiO(IO_3_) followed by their endothermic decomposition at higher temperatures (*i.e.*, 400–600 °C). Similar results had previously been reported for Al_2_O_3_ reacting with AIH decomposition species. The results from this study provide additional information on reaction kinetics between AIH and metal oxides that could be useful for generating multiple reaction pathways for the formation of oxidizers previously shown to have tremendous biocidal potential.

## Conflicts of interest

There are no conflicts to declare.

## Supplementary Material

RA-010-D0RA02250K-s001
