# Supplementary material for: Synthesis of metal iodates from an energetic salt
Source: RSC Adv. 2020 Apr 7;10(24):14403–9. doi: 10.1039/d0ra02250k (PMC9051928; doi:10.1039/d0ra02250k)
Supplement: RA-010-D0RA02250K-s001 [file RA-010-D0RA02250K-s001.pdf]

Electronic Supplementary Material (ESI) for RSC Advances.
This journal is © The Royal Society of Chemistry 2020

Synthesis of Metal Iodates from an Energetic Salt

I. Shancita[1], Kelsea K. Miller[1], Preston D. Silverstein[2],

Joseph Kalman[2], Michelle L. Pantoya[1]*

[1] Department of Mechanical Engineering, Texas Tech University, Lubbock, TX 79409, USA

[2] Mechanical and Aerospace Engineering Department, California State University, Long Beach, CA 90840, USA

Supplementary Information

Table of Contents

S1. XRD spectra of product species from annealed AIH + CuO mixture at indicated Temperatures.......... Page 2

S2. XRD spectra of product species from annealed AIH + Fe_2O_3 mixture at indicated Temperatures .... Page 2

S3. XRD spectra of product species from annealed AIH + Bi_2O_3 mixture at indicated Temperatures .... Page 3

* Corresponding authors:
Email address: michelle.pantoya@ttu.edu (M.L. Pantoya)

S1. XRD spectra of product species from annealed AIH + CuO mixture at indicated temperatures

Figure S1 (a) and **(b)** represent the PXRD scans of product species from annealed AIH + CuO at 360°C and 800°C respectively.

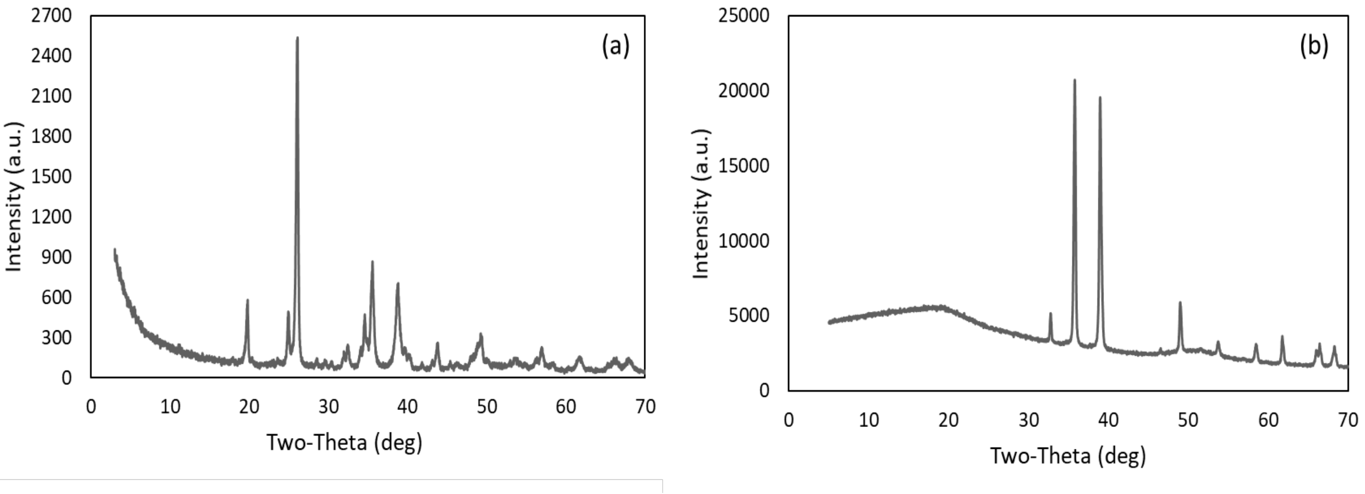

Figure S1. PXRD pattern of identified product species from AIH + CuO mixture annealed at (a) 360°C and (b) 800°C

S2. XRD spectra of product species from annealed AIH + Fe_2O_3 mixture at indicated temperatures

Figure S2 (a) and **(b)** represent the PXRD scans of product species from annealed AIH + Fe_2O_3 at 360°C and 800°C respectively.

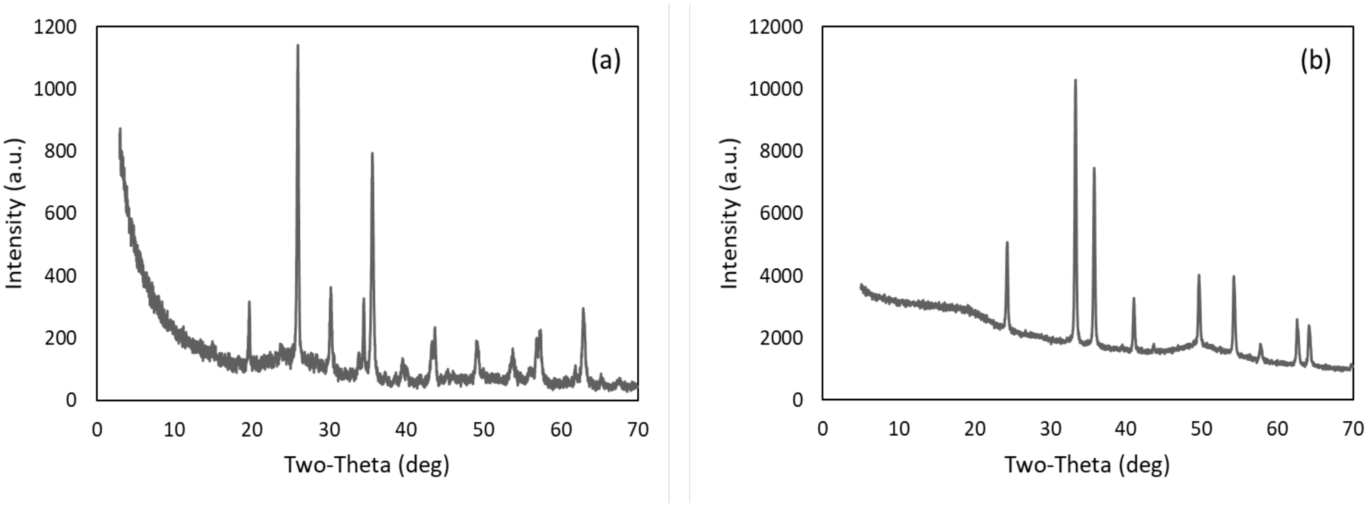

Figure S2. PXRD pattern of identified product species from AIH + Fe_2O_3 mixture annealed at (a) 360°C and (b) 800°C

S3. XRD spectra of product species from annealed AIH + Bi_2O_3 mixture at indicated temperatures

Figure S3 (a) and **(b)** represent the PXRD scans of product species from annealed AIH + Bi_2O_3 at 420°C and 800°C respectively.

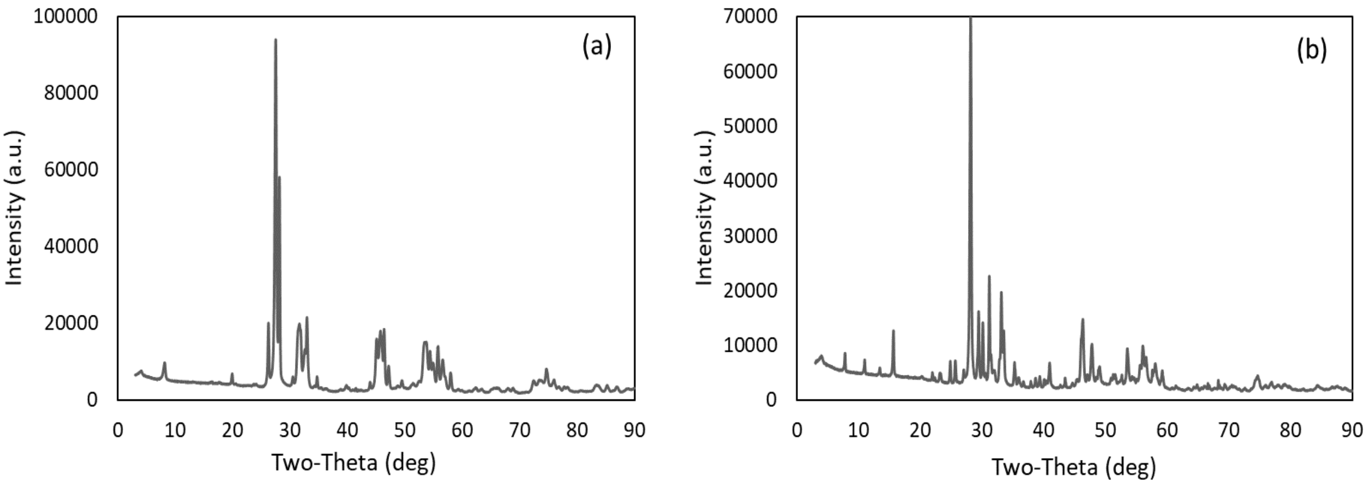

Figure S3. PXRD pattern of identified product species from AIH + Bi_2O_3 mixture annealed at (a) 420°C and (b) 800°C
